# Understanding Farm Households’ Tolerance Toward Asian Elephants in China: Evidence from 873 Households in Yunnan Province

**DOI:** 10.3390/ani16081195

**Published:** 2026-04-14

**Authors:** Junfeng Chen, Yi Xie, Mengyuan Zhang, Weiming Lin, Jie Yang

**Affiliations:** 1 School of Agricultural Economics and Rural Development, Renmin University of China, Beijing 100872, China; junfengchen@ruc.edu.cn; 2 School of Economics and Management, Beijing Forestry University, Beijing 100083, China; yixie@bjfu.edu.cn (Y.X.); zmy21703@163.com (M.Z.); 3 School of Economics and Management, Fujian Agriculture and Forestry University, Fuzhou 350002, China

**Keywords:** Asian elephant conservation, human–elephant conflict, farm households’ tolerance toward Asian elephants, human–elephant coexistence

## Abstract

Improved conservation efforts in China have contributed to the recovery and range expansion of the Asian elephant, increasing spatial overlap between humans and elephants and intensifying the pressure of human–elephant coexistence. Understanding human tolerance toward Asian elephants has therefore become an important pathway for promoting conservation and coexistence. Using survey data from 873 farm households collected in Xishuangbanna Prefecture and Pu’er City in Yunnan Province, this study measures tolerance toward Asian elephants across five dimensions: types of elephant-related damage, economic loss, population size, spatial distance, and activity frequency. Independent-sample *t*-tests, one-way ANOVA and ANCOVA were used to examine differences in tolerance among different groups of farm households. The results show that: (1) the overall tolerance toward Asian elephants among farm households is relatively low (mean = 2.40); (2) within types of elephant-related damage, tolerance is lowest for crop loss and cash crop loss, followed by loss of working time and risk of human injury, while tolerance is relatively higher for damage to houses and property, loss of stored food, and damage to vehicles; (3) farm households showed low levels of tolerance across the dimensions of economic loss, population size, spatial distance, and activity frequency; (4) higher tolerance is observed among male respondents, Dai farm households, those engaged in wildlife protection-related occupations, and households located in Pu’er City, and tolerance increased as cultivated land area decreased, household income increased, and agricultural dependence declined. These findings provide empirical evidence for understanding farm households’ tolerance toward Asian elephants and offer policy insights for improving tolerance, optimizing human–elephant conflict (HEC) management, and promoting the social sustainability of human–elephant coexistence. The study also contributes China-based evidence to discussions on flagship species conservation and community coexistence under the SDG 15 and CBD frameworks.

## Simple Summary

Increasing spatial overlap between humans and Asian elephants has intensified the pressure of human–elephant coexistence. Farm households’ tolerance toward Asian elephants is therefore a key social foundation for effective conservation and long-term coexistence. This study draws on household survey data from Asian elephant distribution areas in Yunnan Province, China. It measures farm households’ tolerance across five dimensions: types of elephant-related damage, economic loss, population size, spatial distance, and activity frequency. Differences in tolerance among farm household groups are also examined. The study provides insights into farm households’ tolerance toward Asian elephants and offers implications for improving human–elephant conflict management and promoting long-term human–elephant coexistence.

## 1. Introduction

Terrestrial biodiversity conservation and ecosystem restoration are key priorities under Sustainable Development Goal 15 (SDG 15) and the Convention on Biological Diversity (CBD). The Asian elephant (*Elephas maximus*), a flagship and umbrella species, plays an important role in maintaining forest ecosystem structure and biodiversity [1,2]. In recent years, strengthened protection policies in China have supported the recovery and range expansion of Asian elephant populations in Yunnan Province. At the same time, intensified land use, agricultural expansion, and infrastructure development have increased spatial overlap between human activities and Asian elephant habitats [3,4,5]. As a result, conservation gains have coincided with rising livelihood costs for local communities in some areas [6,7]. Human–Elephant Conflict (HEC) has therefore become increasingly frequent in rural regions and is now recognized as a key social challenge affecting the long-term sustainability of conservation outcomes [8,9].

For farm households in HEC areas, the impacts of Asian elephants entering farmland and settlements are multidimensional. These impacts include crop loss, cash crop loss, loss of working time, damage to houses and property, loss of stored food, damage to vehicles, and the risk of human injury [10,11,12]. Such direct and indirect costs can shape farm households’ perceptions of Asian elephant conservation and their support for related management measures. To mitigate HEC, the Chinese government and local authorities have implemented various measures, including elephant-proof fencing, the establishment of food-source areas, monitoring and early-warning systems, and patrol management [6,13,14]. As management practices have evolved, conservation agencies have increasingly recognized that tolerance toward Asian elephants among farm households is a critical social foundation influencing conservation outcomes and the stability of human–elephant coexistence [15,16]. Previous studies also suggest that the persistence of wildlife in human-dominated landscapes depends not only on ecological carrying capacity but also on human behavior [17]. A key determinant of such behavior is human tolerance toward the species concerned [18]. When ecological carrying capacity cannot be substantially altered in the short term, increasing human tolerance becomes an important pathway for promoting human–elephant coexistence [19]. Therefore, identifying and understanding the tolerance toward Asian elephants among farm households in Asian elephant distribution areas, as well as its variation across different groups, is of considerable theoretical and practical importance.

Research on wildlife tolerance has received increasing attention in recent years and has become an important perspective for understanding human–wildlife conflict. Existing studies can generally be grouped into two strands. The first focuses on psychological and attitudinal perspectives [20]. In this line of research, tolerance is often defined as people’s attitudes toward the presence and behavior of wildlife, or the threshold at which wildlife impacts remain acceptable. These studies typically examine public acceptance of wildlife conservation, population changes, and related management measures [21,22,23]. The second strand emphasizes economic and livelihood perspectives. Here, tolerance is understood as the willingness and capacity of people to bear wildlife-related losses and risks under coexistence conditions. Such studies pay particular attention to the effects of economic loss, livelihood impacts, and compensation mechanisms on human attitudes toward wildlife [24,25,26]. In terms of measurement, early research often relied on single attitudinal items to assess human tolerance toward wildlife. However, this approach has limitations. It struggles to explain the common “attitude–behavior inconsistency” and provides limited guidance for conflict management [27,28,29]. As the field has developed, researchers have increasingly adopted multidimensional measurement frameworks. These approaches incorporate factors such as loss experiences, behavioral intentions, preferences regarding population change, acceptable spatial distance, and support for management measures. Such frameworks allow a more comprehensive assessment of the acceptable boundaries of human–wildlife coexistence and improve both explanatory power and policy relevance [30,31,32].

Although research on wildlife tolerance has grown rapidly, several gaps remain. First, empirical evidence on certain species and contexts is still limited. Much of the existing literature focuses on large carnivores such as wolves, bears, and leopards, while relatively little research has examined Asian elephants, particularly using farm households in Asian elephant distribution areas of China as the unit of analysis. Second, measurement approaches remain relatively limited. Some studies still rely primarily on single attitudinal items or a small number of behavioral intention indicators. Such approaches make it difficult to translate tolerance into specific information useful for policy design. For example, they rarely capture how farm households differ in their tolerance toward different types of elephant-related damage, economic loss, spatial distance, or activity frequency. Third, the role of social heterogeneity remains insufficiently explored. Effective management of HEC relies heavily on local social support. However, farm households differ substantially in sociodemographic characteristics, income levels, livelihood structures, and spatial contexts. Without recognizing this heterogeneity, mitigation measures, compensation arrangements, and risk management tools may fail to match the needs of different groups, thereby reducing both policy effectiveness and perceived fairness.

Against this background, this study develops a multidimensional measurement framework of tolerance toward Asian elephants at the farm household level to better address the practical needs of human–elephant coexistence. The analysis is based on survey data from 873 farm households collected through field investigations in Xishuangbanna Prefecture and Pu’er City, Yunnan Province, China. This study focuses on three main issues. First, it examines the overall level of farm households’ tolerance toward Asian elephants in elephant distribution areas. Second, it analyzes the level and structural features of tolerance across five dimensions: types of elephant-related damage, economic loss, population size, spatial distance, and activity frequency. Third, it tests differences in tolerance among farm households with different individual and household characteristics. The findings provide targeted evidence for improving risk mitigation, compensation mechanisms, and livelihood support measures, as well as for strengthening community participation and conservation collaboration. The study also contributes China-based evidence to discussions on long-term human–elephant coexistence governance under the SDG 15 and CBD frameworks.

## 2. Materials and Methods

### 2.1. Study Area

**Figure 1:**
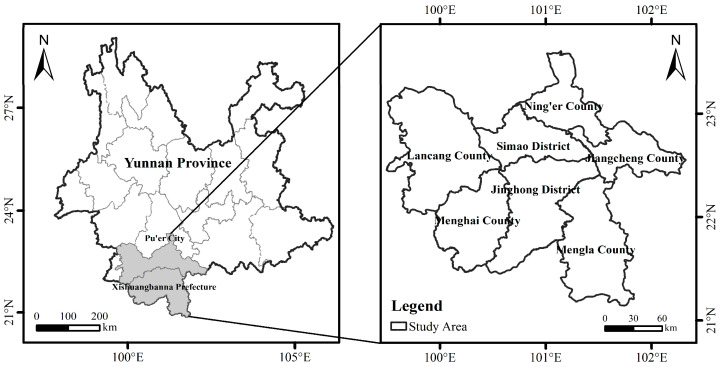
Map of the study area.

Yunnan Province is currently the only region in China where wild Asian elephants are found [33]. Recent monitoring and surveys estimate that the Asian elephant population in Yunnan is about 300 individuals [6]. Xishuangbanna Prefecture and Pu’er City constitute the core habitats and primary activity areas of Asian elephants, accounting for more than 95% of the total population [34]. Xishuangbanna has historically been the primary habitat of Asian elephants. In recent years, however, their activity range has gradually expanded from Xishuangbanna toward Pu’er [35,36]. Based on the main distribution areas of Asian elephants, this study selects Xishuangbanna Prefecture (Jinghong City, Mengla County, and Menghai County) and Pu’er City (Jiangcheng County, Lancang County, Ning’er County, and Simao District) as the study area, providing a representative context for analysis (Figure 1). The spatial distribution map of the study area was generated using ArcGIS 10.8 (Esri, Redlands, CA, USA).

### 2.2. Data Collection

Fieldwork was conducted in Yunnan Province from July to August 2023. The survey team consisted of graduate students, faculty members, and local staff engaged in wildlife protection, all of whom had prior survey experience. Before the formal survey, all investigators received standardized training on questionnaire administration to ensure interview consistency and data quality. A combination of purposive and random sampling was used. Surveys were conducted in counties (districts/cities) where Asian elephants are distributed in Xishuangbanna Prefecture and Pu’er City. Based on the distribution of Asian elephants and recommendations from local wildlife management authorities, one to two townships were selected in each county (district/city). Within each township, two to four administrative villages were randomly chosen, and 20–30 farm households were randomly sampled in each village. The questionnaire survey was conducted through face-to-face interviews. Investigators spoke directly with respondents and recorded their answers. The questionnaire was completed by the household head or the primary decision-maker, representing the attitudes of the entire farm household. In total, 873 valid questionnaires were obtained.

### 2.3. Variable Selection

Because tolerance has a strong subjective dimension, this study measures the perceived acceptability of human–elephant coexistence among farm households under existing compensation and management conditions, rather than directly assessing objective risk exposure or the actual intensity of damage [37,38]. It should also be noted that tolerance is not merely an emotional attitude. Instead, it reflects a comprehensive judgment formed by farm households when weighing the costs and risks associated with coexistence. Based on this understanding, this study defines farm households’ tolerance toward Asian elephants in the following way. It refers to the attitudinal response of farm households to the extent to which they can bear the risks, losses, and coexistence constraints associated with living alongside Asian elephants [39,40]. Drawing on previous research and typical HEC situations in Asian elephant distribution areas of Yunnan Province, this study develops a multidimensional measurement framework of tolerance toward Asian elephants at the farm household level [6,36,41]. The framework aims to translate the abstract concept of tolerance into empirical indicators that can be decomposed and compared. Based on a pilot survey and field feedback, the wording of questionnaire items and response options was further refined to improve clarity and contextual relevance.

This study measures farm households’ tolerance toward Asian elephants across five dimensions: types of elephant-related damage, economic loss, population size, spatial distance, and activity frequency. These dimensions correspond to key aspects of HEC management, including consequences, economic impacts, population scale, spatial proximity, and activity frequency [7,33]. This design allows a multidimensional assessment of the acceptable range of human–elephant coexistence among farm households, while avoiding reliance on a single indicator to represent the complex structure of tolerance. The specific measurement items are presented in Table 1.

### 2.4. Research Methods

Tests of group differences were used to identify variations in tolerance toward Asian elephants among farm households [42,43]. First, a one-sample *t*-test was conducted to examine whether the mean values of each item or dimension significantly deviated from the midpoint of the scale (3 = neutral). Second, differences in tolerance among different groups of farm households were examined. Independent-sample *t*-tests were applied to binary variables, including gender, whether respondents were engaged in wildlife protection-related occupations, and region. For variables with multiple categories—age, education level, ethnicity, cultivated land area, household income, and agricultural dependence—one-way ANOVA was used. The comparisons covered five dimensions: types of elephant-related damage, economic loss, population size, spatial distance, and activity frequency, as well as the overall tolerance index. To ensure the robustness of the results, we further conducted an ANCOVA. Age, cultivated land area, household income, and agricultural dependence were included as covariates. The effects of gender, ethnicity, education level, wildlife protection-related occupation, and region on overall tolerance were then examined. All data processing and statistical analyses were conducted using IBM SPSS Statistics 26.0 (IBM Corp., Armonk, NY, USA).

## 3. Results

### 3.1. Tolerance Toward Different Types of Elephant-Related Damage

**Figure 2:**
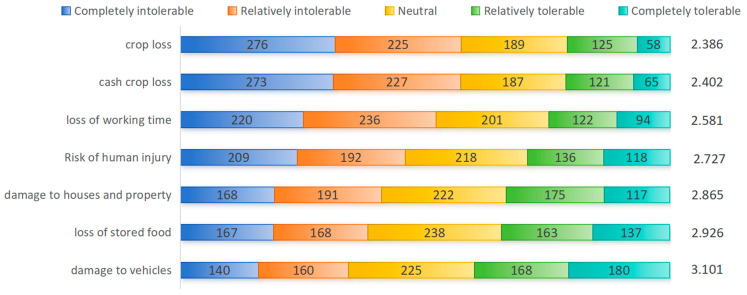
Distribution of Farm Households’ Tolerance toward Types of Elephant-Related Damage (Unit: households). Note: The right side of each bar shows the mean value for that dimension.

The distribution of farm households’ tolerance toward different types of elephant-related damage is presented in Figure 2. Overall, tolerance toward crop loss and cash crop loss shows a highly similar pattern and is clearly concentrated at low tolerance levels. Specifically, 57.39% and 57.27% of farm households reported that these losses were “intolerable,” while only 20.96% and 21.31% considered them “tolerable.” These results indicate that farm households are particularly sensitive to damage affecting key agricultural crops. In comparison, tolerance toward loss of working time is also relatively low. About 52.23% of farm households reported that such losses were “intolerable,” while 24.74% considered them “tolerable.” This finding suggests that the loss of working time and the associated opportunity costs caused by Asian elephants also have a substantial impact on farm households.

Tolerance toward the risk of human injury shows a pattern characterized by predominantly low tolerance, while the proportion of neutral responses remains relatively high. Specifically, 401 farm households (45.93%) reported low tolerance, and the largest share, 218 households (24.97%), selected the neutral option. This suggests that although most farm households are unwilling to tolerate the risk of human injury caused by Asian elephants, a considerable proportion remain neutral or uncertain in their attitudes.

In comparison, tolerance toward damage to houses and property and loss of stored food is closer to the midpoint of the scale, and the two dimensions display similar distribution patterns. The proportion of low tolerance is 36.90% and 38.37%, respectively, which is more than ten percentage points lower than that observed for crop loss, cash crop loss, and loss of working time. Meanwhile, 22.82% and 27.26% of respondents reported neutral attitudes, while 30.01% and 34.36% indicated that these losses were tolerable. Overall, tolerance toward these two types of damage is relatively higher. Among them, loss of stored food is closest to the midpoint of the scale and reflects a moderate level of tolerance.

Among all types of damage, tolerance toward damage to vehicles is the highest. A total of 348 farm households (39.86%) reported that such losses were tolerable, which is higher than the 300 households (34.36%) who considered them intolerable. Overall, tolerance toward this type of damage has reached or slightly exceeded the midpoint of the scale.

Taken together, the results reveal a clear gradient across different types of elephant-related damage, with tolerance increasing in the following order: crop loss, cash crop loss, loss of working time, risk of human injury, damage to houses and property, loss of stored food, and damage to vehicles. This pattern indicates that losses directly related to agricultural production and immediate livelihood impacts are the least tolerable. In contrast, damages related to household assets or stored goods are relatively more acceptable to farm households.

To examine whether the mean values of each item significantly deviated from the midpoint of the scale (3 = neutral), a one-sample *t*-test was conducted for all items under types of elephant-related damage (test value = 3). The results are reported in Table 2. Except for loss of stored food, most items show mean values that differ significantly from 3 and are generally below the moderate tolerance level. Specifically, tolerance toward crop loss, cash crop loss, loss of working time, and risk of human injury is significantly lower than the neutral level, with mean values of 2.386, 2.402, 2.581, and 2.727, respectively (all *p* < 0.001). Tolerance toward damage to houses and property is also significantly below 3 (mean = 2.865, *p* = 0.002), although the deviation from the midpoint is relatively small. In contrast, the item loss of stored food does not reach statistical significance (mean = 2.926, *p* = 0.098), indicating that its tolerance level is not significantly different from the scale midpoint and is therefore close to a neutral level. Notably, the mean value for damage to vehicles is slightly above 3 and statistically significant (mean = 3.101, *p* = 0.028). This suggests that farm households show relatively higher tolerance toward this type of elephant-related damage, although the overall level remains close to moderate tolerance.

### 3.2. Farm Households’ Tolerance Across Other Dimensions

Tolerance toward Asian elephants was further examined across four dimensions: economic loss, population size, spatial distance, and activity frequency. The descriptive statistics are presented in Table 3. Overall, tolerance levels are relatively low across all dimensions, although some variation exists among them.

For the economic loss dimension, tolerance is generally low and strongly concentrated at the lower end of the scale. A total of 66.09% of farm households reported either “very low” or “low” tolerance, including 43.87% indicating “very low.” In contrast, 26.57% reported “high” or “very high” tolerance. The mean value for this dimension is 2.26, clearly below the scale midpoint of 3, suggesting that farm households have limited tolerance toward the economic loss caused by Asian elephants.

For population size, tolerance also remains relatively low. The proportion of farm households reporting “very low” or “low” tolerance is 57.27%, while 16.72% reported a neutral level and 26.00% reported “high” or “very high” tolerance. The mean value for this dimension is 2.46, which is below the scale midpoint. This indicates that farm households in the sample generally show limited tolerance toward the current population size of nearby Asian elephants.

For spatial distance, the acceptable level of proximity between humans and elephants is also relatively low. The proportion of low tolerance reaches 50.57%, including 33.92% reporting “very low” tolerance, while 22.41% report “high” or “very high” tolerance. The mean value of 2.34 suggests that most farm households prefer a greater spatial distance between Asian elephant activity areas and residential areas.

Among the four dimensions, tolerance toward activity frequency is the lowest. A total of 72.86% of farm households report low tolerance, including 43.99% indicating “low” and 28.87% indicating “very low.” Only 22.57% report “high” or “very high” tolerance. The mean value for this dimension is 2.24, indicating that most farm households do not want Asian elephants to remain frequently active in their local areas.

To examine whether the mean values of the four dimensions significantly deviate from the midpoint of the scale (test value = 3), a one-sample *t*-test was conducted (Table 4). The results show that the mean values for economic loss, population size, spatial distance, and activity frequency are all significantly lower than 3 (all *p* < 0.001), and the mean differences are negative in each case. These findings indicate that, at the overall attitudinal level, farm households exhibit tolerance levels significantly below the moderate level in terms of acceptable economic loss, acceptable population size, acceptable spatial distance, and acceptable activity frequency of Asian elephants. Considering all five dimensions together, the overall mean level of tolerance toward Asian elephants among farm households is 2.40.

### 3.3. One-Way ANOVA of Tolerance Based on Individual and Household Characteristics

#### 3.3.1. Gender Differences

As shown in Table 5, significant gender differences were found across all five dimensions and overall tolerance (all *p* < 0.001). Overall, farm households represented by male respondents report higher tolerance toward Asian elephants than those represented by female respondents across all dimensions and in overall tolerance. The differences are particularly pronounced for population size and spatial distance tolerance.

#### 3.3.2. Age Differences

As shown in Table 6, although mean values across different age groups vary slightly across all dimensions and overall tolerance, the results of the one-way ANOVA do not reach statistical significance. This indicates that no stable differences in tolerance toward Asian elephants are observed among different age groups in this sample. From the distribution of mean values, the young group (18–30 years) shows relatively higher tolerance in terms of economic loss, population size, spatial distance, activity frequency, and overall tolerance compared with other age groups.

#### 3.3.3. Education Level Differences

As shown in Table 7, the group with college or above showed relatively higher mean values across most dimensions. However, differences across education groups were generally not statistically significant for any dimension or for overall tolerance.

#### 3.3.4. Ethnic Differences

As shown in Table 8, significant differences across ethnic groups are observed for all five dimensions and overall tolerance toward Asian elephants (all *p* < 0.001). The results indicate that farm households represented by Dai respondents report the highest mean values across all dimensions and in overall tolerance (overall tolerance = 3.11), whereas farm households represented by Han respondents show the lowest levels of tolerance (overall tolerance = 1.85). Tolerance levels among Hani, Lahu, and other ethnic minority households fall between these two groups. These findings suggest that ethnicity is an important factor explaining the heterogeneity in farm households’ tolerance toward Asian elephants.

#### 3.3.5. Occupational Differences

As shown in Table 9, significant differences are observed across all dimensions and overall tolerance toward Asian elephants between respondents engaged in wildlife protection-related occupations (Asian elephant monitors and forest rangers) and those who are not (all *p* < 0.001). Farm households represented by respondents working in wildlife protection-related occupations report significantly higher tolerance levels across all dimensions and in overall tolerance compared with those not engaged in such occupations. The magnitude of these differences is also relatively large. These results suggest that farm households involved in wildlife protection-related work generally exhibit higher tolerance toward Asian elephants.

#### 3.3.6. Regional Differences

Table 10 compares the regional differences in tolerance toward Asian elephants between farm households in Xishuangbanna Prefecture and Pu’er City. The results show significant regional differences across all dimensions. In terms of mean values, farm households in Pu’er City report higher tolerance levels than those in Xishuangbanna Prefecture across all dimensions and in overall tolerance.

#### 3.3.7. Differences by Cultivated Land Area

Table 11 compares the differences in tolerance toward Asian elephants among farm households with different cultivated land areas. The results showed that mean tolerance increased as cultivated land area decreased. In terms of statistical significance, only tolerance toward spatial distance showed a significant difference across groups (*p* = 0.011), indicating that households with larger cultivated land areas had lower mean tolerance toward spatial distance.

#### 3.3.8. Differences by Household Income

Table 12 presents the differences in tolerance toward Asian elephants across household income groups. The results show that significant differences exist across all five dimensions and in overall tolerance (all *p* < 0.001). Overall, mean tolerance toward Asian elephants tends to increase as household income rises.

#### 3.3.9. Differences by Agricultural Dependence

Table 13 shows the differences in tolerance toward Asian elephants across groups with different levels of agricultural dependence. Significant differences are observed across all dimensions and in overall tolerance. Based on the mean values, tolerance decreases as agricultural dependence increases. Farm households with agricultural dependence ≤ 20% report the highest tolerance across all dimensions, including types of elephant-related damage, economic loss, population size, spatial distance, activity frequency, and overall tolerance (overall mean = 2.81). In contrast, farm households with agricultural dependence of 80–100% report the lowest tolerance levels (overall mean = 2.08).

### 3.4. ANCOVA of Tolerance Based on Individual and Household Characteristics

To further examine differences in overall tolerance across groups and ensure the robustness of the results, this study conducted an ANCOVA in addition to independent-samples *t*-tests and one-way ANOVA. As shown in Table 14, the overall model was statistically significant, with an adjusted R^2^ of 0.364. This indicates that ethnicity, gender, education, occupation, region, age, cultivated land area, agricultural dependence, and household income jointly explain variations in overall tolerance among farm households.

After controlling for age, cultivated land area, agricultural dependence, and household income, ethnicity, gender, occupation, and region remained significantly associated with overall tolerance, whereas education was not significant. Ethnicity showed the strongest effect, followed by region and occupation. This suggests that differences in tolerance related to ethnicity, region, and occupation remained relatively robust after controlling for key continuous variables. Parameter estimates further showed that male respondents, Dai households, households engaged in wildlife protection-related occupations, and households located in Pu’er had higher overall tolerance.

Further Bonferroni pairwise comparisons based on estimated marginal means showed that the ethnic differences were mainly reflected in the significantly higher overall tolerance of Dai households than that of all other groups, while Han households showed significantly lower overall tolerance than all other groups. Differences among the remaining ethnic groups were not statistically significant.

Among the covariates, cultivated land area, agricultural dependence, and household income all had significant effects on overall tolerance, whereas age was not significant. Specifically, overall tolerance decreased as cultivated land area increased and as agricultural dependence rose. By contrast, overall tolerance increased as household income increased. The corresponding parameter estimates and Bonferroni pairwise comparison results for ethnicity are provided in the Appendix A.

## 4. Discussion

### 4.1. Tolerance Gradient Across Types of Elephant-Related Damage and Its Livelihood Relevance

Across the dimension of types of elephant-related damage, farm households show a clear gradient in tolerance toward Asian elephants. Tolerance levels increase in the following order: crop loss, cash crop loss, loss of working time, risk of human injury, damage to houses and property, loss of stored food, and damage to vehicles. This pattern indicates that farm households are most sensitive to conflicts that occur frequently and directly affect agricultural production and household livelihood security [44]. In particular, crop loss not only occurs more often but is also closely linked to seasonal income and household food security. As a result, it is more likely to generate strong negative perceptions and is associated with the lowest tolerance levels [45,46].

Tolerance toward loss of working time is also relatively low, suggesting that farm households are sensitive to the opportunity costs associated with time and labor constraints. Compared with crop loss, this type of damage represents a more indirect cost, as it mainly affects household income by reducing time available for agricultural work or off-farm employment [47]. However, current wildlife damage compensation only covers direct economic losses, such as crop loss and human injury, and provides no financial compensation for indirect losses such as loss of working time. Although such losses are less visible than crop damage, their economic effects can accumulate over time, making them similarly difficult for farm households to accept. In other words, low tolerance arises not only from visible losses, such as damaged crops, but also from less visible costs, including lost labor time and opportunity costs [48].

By contrast, tolerance toward risk of human injury, damage to houses and property, and loss of stored food remains relatively low overall but is noticeably higher than for crop loss and loss of working time. These values are also closer to the midpoint of the scale. This pattern may reflect the lower frequency of such events and the heterogeneity of individual experiences. Some farm households may not have directly encountered these incidents or may have experienced them only to a limited extent, leading to more neutral responses [49]. In particular, the relatively high proportion of neutral responses regarding risk of human injury suggests that some respondents recognize the existence of the risk but have not yet formed a strong or stable attitude toward it. This may be explained by the relatively low number of human injury incidents caused by Asian elephants in the study area.

Tolerance toward damage to vehicles is the highest among all conflict types. This may be because such incidents are relatively rare in the daily lives of farm households or because their consequences can often be addressed through repair, replacement, or external assistance. As a result, the direct impact on long-term household livelihoods is comparatively limited [50]. It should be noted, however, that higher tolerance does not imply that this type of conflict is unimportant. Rather, it indicates that it occupies a lower priority in farm households’ overall risk perception.

### 4.2. Key Dimensions of Tolerance in Human–Elephant Coexistence

Across the four dimensions of economic loss, population size, spatial distance, and activity frequency, tolerance levels are all significantly below the midpoint of the scale. This suggests that the acceptable boundary of coexistence is influenced not only by the types of elephant-related damage, but also by the frequency of conflict events and the accumulation of economic losses [51]. When Asian elephants appear repeatedly and cause frequent disturbances, farm households may become increasingly intolerant even if the loss from any single event is relatively small [52]. This finding indicates that, in the daily experience of farm households, HEC is not merely an isolated loss event but rather a continuous disturbance process. Frequent elephant activity not only increases the risk of crop loss, but may also disrupt daily farming schedules, affect travel safety, and heighten psychological uncertainty about future risks [53]. Moreover, when economic losses accumulate through repeated conflict events, farm households are more likely to develop negative attitudes toward elephant presence [48]. Therefore, in efforts to promote human–elephant coexistence, management strategies should not only aim to reduce individual loss events but also focus on lowering the frequency of elephant incursions into farmland or residential areas, while strengthening compensation mechanisms to reduce the actual economic burden on farm households and mitigate their perception of persistent conflict risks [51,54].

Tolerance toward spatial distance is also relatively low, reflecting farm households’ tendency to avoid the safety risks and uncertainty associated with close human–elephant proximity. As the spatial distance between elephants and human settlements decreases, the unpredictability of potential conflicts increases, and daily production and livelihood activities are more likely to be disrupted, thereby lowering tolerance levels [55,56]. For example, when elephant activity occurs near farmland or villages, farm households often need to devote additional time to monitoring, guarding, or adjusting farming practices. This not only increases labor costs but also reinforces their perception of ongoing risk.

The mean tolerance toward population size is also below the midpoint of the scale. This suggests that Asian elephant population size is as easy to perceive as elephant visitation frequency, proximity to residential areas, and the economic losses caused. All of these serve as important cues shaping farm households’ risk perception and judgments about coexistence [57,58]. This finding implies that perceptions of coexistence are not based on a single dimension. Instead, they reflect the combined influence of economic loss, population size, activity frequency, and spatial distance. Therefore, efforts to enhance tolerance should adopt a more targeted approach and address multidimensional risks. In particular, spatial management and risk prevention measures should be strengthened. These measures can reduce the frequency of elephant incursions into farmland and residential areas. They can also lessen the economic losses and disruptions to daily life caused by conflict. In this way, they may help alleviate the risk perception and uncertainty associated with close human–elephant proximity [19,59].

### 4.3. Differences in Tolerance Across Socio-Demographic and Household Characteristics

Tolerance toward Asian elephants shows clear heterogeneity across socio-demographic and household characteristics, although these differences are not consistent across all variables. This suggests that tolerance is shaped by specific socio-economic contexts rather than by any single factor. At the individual level, gender, ethnicity, and engagement in wildlife protection-related occupations are significantly associated with tolerance. In particular, Dai farm households show significantly higher tolerance than all other ethnic groups. This may be closely related to local cultural identity and traditional beliefs [60,61]. In Dai culture, elephants are widely regarded as symbols of good fortune and agricultural prosperity, and they occupy an important place in religious practices and traditional customs. As a result, farm households may be more likely to adopt a positive or tolerant attitude toward the presence of Asian elephants [62].

In addition, farm households represented by respondents engaged in wildlife protection-related occupations also show higher tolerance levels. This may partly reflect their greater awareness of conservation policies and ecological protection values, as well as their involvement in conservation practices and potential benefits derived from them [63,64]. Female farm households show relatively lower tolerance toward Asian elephants, which may be related to women’s greater involvement in farming activities around the home and in daily agricultural production. Because women more frequently come into direct contact with farmland and situations involving crop loss, they may perceive elephant-related crop loss and disruptions to daily production more directly, which may contribute to lower tolerance. Overall, these findings indicate that differences in tolerance are more closely related to local social structures, cultural contexts, and the degree of engagement with conservation activities, rather than simply determined by generational or educational differences [35,65].

In terms of region, farm households in Pu’er City show higher overall tolerance than those in Xishuangbanna Prefecture. This difference may reflect variations in both conflict exposure and governance contexts between the two regions. On the one hand, Xishuangbanna is the traditional habitat of Asian elephants in China, where HEC have occurred for a longer period and at higher frequency. Long-term exposure to such conflicts may increase risk perceptions among farm households and reduce their tolerance toward elephant activity [35,66,67]. On the other hand, Pu’er has recently invested more resources in wildlife damage compensation programs, with relatively higher compensation standards and payment levels. When HEC occur, higher compensation may partially offset actual economic losses [68]. Because farm households tend to be particularly sensitive to economic loss, more adequate compensation mechanisms may help reduce negative perceptions associated with conflicts and increase tolerance toward Asian elephants [69,70]. This finding is consistent with previous studies suggesting that compensation or benefits derived from conservation can increase tolerance toward wildlife [71,72].

Regarding household income, tolerance increases with rising household income, indicating that greater economic capacity can help buffer the financial risks associated with HEC [73]. When household income sources become more diversified and dependence on agricultural income declines, farm households are typically better able to absorb wildlife-related losses and therefore tend to show higher tolerance levels [74].

In terms of agricultural dependence, tolerance toward Asian elephants decreases as reliance on agricultural income increases. This indicates that when farm households depend heavily on agriculture and have limited alternative income sources, HEC impose a stronger marginal impact on their livelihoods, thereby reducing tolerance. For households whose primary income derives from agriculture, elephant activity in farmland can cause direct crop loss while also increasing uncertainty in agricultural production, further heightening sensitivity to livelihood risks [36,75]. By contrast, households with lower agricultural dependence often have more diversified income sources, including off-farm employment, which provides greater resilience against wildlife-related losses and is associated with higher tolerance levels [76]. Overall, these results highlight the importance of livelihood structure in shaping tolerance toward wildlife in HEC areas.

Finally, the effect of cultivated land area on tolerance is mainly reflected in the dimensions of spatial distance. In general, farm households with larger cultivated land areas show lower tolerance for elephants approaching farmland. This may be because larger farmland areas increase the spatial exposure of agricultural production, meaning that when elephants enter farmland, the potential scale of damage and economic loss is greater [77,78]. At the same time, larger farmland areas may increase the likelihood of spatial overlap between elephant activity and agricultural land, thereby raising the probability and frequency of elephant incursions into farmland and reinforcing farmers’ sensitivity to such risks [79,80]. Consequently, in addition to the size of farmland itself, farm households are particularly concerned with the distance between farmland and elephant activity areas.

### 4.4. Research Limitations

This study has several limitations. First, the questionnaire relies on self-reported responses, which may be influenced by individual risk perceptions and social desirability, potentially introducing some bias. Second, the analysis mainly employs descriptive statistics, one-sample tests, and group comparison tests. While these methods help identify the overall level of tolerance and differences across farm household groups, they provide limited insight into the underlying mechanisms influencing tolerance. Future research could further explore the determinants and causal mechanisms of farm households’ tolerance toward Asian elephants.

## 5. Conclusions

This study finds that farm households show relatively low overall tolerance toward Asian elephants. In terms of types of elephant-related damage, losses directly related to agricultural production and household livelihood security are the least tolerated. In addition, tolerance varies significantly across individual and household characteristics. Male respondents, Dai households, households engaged in wildlife protection-related occupations, and households located in Pu’er show relatively higher tolerance. In addition, overall tolerance is also higher among households with smaller cultivated land areas, higher income levels, and lower agricultural dependence. These multidimensional findings suggest that improving the social sustainability of human–elephant coexistence requires addressing the dimensions of conflict that farm households perceive as most sensitive. At the same time, reducing uncertainty and opportunity costs associated with close human–elephant proximity may help enhance conservation outcomes and promote coexistence.

First, priority should be given to reducing livelihood impacts caused by crop loss and loss of working time. As these represent the most intolerable consequences of HEC for farm households, mitigation measures should focus on key stages of agricultural production. In areas with frequent conflict, early-warning systems, patrol management, and protective infrastructure should be strengthened during critical farming seasons and in high-risk farmland areas to reduce the probability of elephants entering farmland. This can help lower the risk of crop damage as well as the time costs associated with protective activities undertaken by farmers. In addition, local agricultural conditions may allow for adjustments in cropping patterns or the introduction of alternative crops, thereby reducing dependence on crops that are particularly vulnerable to elephant damage and mitigating the direct livelihood impacts of conflict.

Second, management efforts should shift from simply reducing losses to reducing the frequency of conflict events. Farm households are relatively sensitive to the activity frequency of Asian elephants, suggesting that compensation or reductions in individual loss events alone may not substantially improve attitudes toward coexistence. Instead, reducing the frequency of elephant incursions into farmland and residential areas, limiting persistent disturbances, and controlling uncertainty associated with conflict risks are likely to be more effective in increasing tolerance. HEC management should therefore move beyond post-event compensation and place greater emphasis on spatial management, early-warning systems, and habitat-based strategies to reduce the ongoing disruptions caused by elephant activity in human-dominated landscapes.

Third, differentiated governance and targeted support for vulnerable groups should be strengthened. Significant differences in tolerance exist across regions, income levels, and degrees of agricultural dependence. As a result, policy design should incorporate context-specific approaches in resource allocation, compensation mechanisms, and risk communication. Particular attention should be given to farm households with lower tolerance levels and more vulnerable livelihoods, such as those with high agricultural dependence or high exposure to conflict. Providing targeted compensation, technical assistance, and risk management support for these groups can improve the effectiveness and fairness of conflict mitigation policies, while avoiding the inefficiencies associated with one-size-fits-all approaches.

## Figures and Tables

**Table 1:** Measurement Items for Tolerance toward Asian Elephants.

Tolerance Dimensions	Variable	Definition	Scale
Types of elephant-related damage	Tolerance toward crop loss	What is your level of tolerance toward crop loss caused by Asian elephants?	1 = Completely intolerable; 2 = Relatively intolerable; 3 = Neutral; 4 = Relatively tolerable; 5 = Completely tolerable
	Tolerance toward cash crop loss	What is your level of tolerance toward cash crop loss caused by Asian elephants?	
	Tolerance toward loss of working time	What is your level of tolerance toward loss of working time (delays in work and farming activities) caused by Asian elephants?	
	Tolerance toward risk of human injury	What is your level of tolerance toward the risk of human injury caused by Asian elephants?	
	Tolerance toward damage to houses and property	What is your level of tolerance toward damage to houses and property caused by Asian elephants?	
	Tolerance toward loss of stored food	What is your level of tolerance toward loss of stored food caused by Asian elephants?	
	Tolerance toward damage to vehicles	What is your level of tolerance toward damage to vehicles caused by Asian elephants?	
Economic loss	Tolerance toward economic loss	Under current compensation and management conditions, what is the maximum annual net economic loss (after compensation) you are willing to tolerate from Asian elephants?	1 = ≤ USD 145 2 = USD 146–291; 3 = USD 292–509; 4 = USD 510–727; 5 = > USD 727
Population size	Tolerance toward population size	From your perspective, what change would you prefer in the local Asian elephant population size in the future?	1 = Decrease significantly 2 = Decrease slightly 3 = Remain unchanged 4 = Increase slightly 5 = Increase significantly
Spatial distance	Tolerance toward spatial distance	What is the minimum distance you consider acceptable between Asian elephant activity areas and residential areas?	1 = ≥ 10 km 2 = 7.5–10 km 3 = 5–7.5 km 4 = 2.5–5 km 5 = ≤ 2.5 km
Activity frequency	Tolerance toward activity frequency	Under current compensation and protection conditions, what is your level of tolerance toward Asian elephants continuing to be active in this area?	1 = Completely intolerable 2 = Relatively intolerable 3 = Neutral 4 = Relatively tolerable 5 = Completely tolerable

**Table 2:** One-Sample *t*-Test Results for Farm Households’ Tolerance toward Types of Elephant-Related Damage.

Types of Elephant-Related Damage	t Value	df	*p* Value	Mean Difference	95% CI (Lower)	95% CI (Upper)	Mean	Standard Error
crop loss	−14.543	872	0.000	−0.614	−0.697	−0.531	2.386	1.247
cash crop loss	−14.004	872	0.000	−0.598	−0.682	−0.514	2.402	1.262
damage to houses and property	−3.055	872	0.002	−0.135	−0.222	−0.048	2.865	1.307
loss of stored food	−1.654	872	0.098	−0.074	−0.163	0.014	2.926	1.330
damage to vehicles	2.199	872	0.028	0.101	0.0108	0.191	3.101	1.354
loss of working time	−6.001	872	0.000	−0.273	−0.362	−0.184	2.581	1.294
risk of human injury	−9.571	872	0.000	−0.419	−0.506	−0.333	2.727	1.342

**Table 3:** Descriptive Statistics of Farm Households’ Tolerance toward Asian Elephants across Other Dimensions.

Tolerance Dimension	Very Low	Low	Neutral	High	Very High	Mean	Std. Dev.
Economic loss	383	194	64	148	84	2.26	1.41
	(43.87%)	(22.22%)	(7.33%)	(16.95%)	(9.62%)		
Population size	275	225	146	154	73	2.46	1.32
	(31.50%)	(25.77%)	(16.72%)	(17.64%)	(8.36%)		
Spatial distance	330	162	163	193	25	2.34	1.26
	(33.92%)	(16.65%)	(16.75%)	(19.84%)	(2.57%)		
Activity frequency	252	384	40	169	28	2.24	1.16
	(28.87%)	(43.99%)	(4.58%)	(19.36%)	(3.21%)		

Note: Percentages are shown in parentheses.

**Table 4:** One-Sample *t*-Test Results for Farm Households’ Tolerance toward Asian Elephants across Other Dimensions.

Tolerance Dimension	t Value	df	*p* Value	Mean Difference	95% CI (Lower)	95% CI (Upper)
Economic loss	−15.452	872	0.000	−0.738	−0.831	−0.644
Population size	−12.206	872	0.000	−0.544	−0.632	−0.457
Spatial distance	−15.516	872	0.000	−0.663	−0.747	−0.580
Activity frequency	−19.595	872	0.000	−0.766	−0.843	−0.690

**Table 5:** Gender Differences in Tolerance toward Asian Elephants.

Tolerance Dimension	Male (*N* = 624)	Female (*N* = 249)	t Value	df	*p* Value
Types of elephant-related damage	2.82 ± 1.06	2.43 ± 1.08	4.932	871	0.000
Economic loss	2.37 ± 1.45	2.00 ± 1.27	3.638	517	0.000
Population size	2.60 ± 1.34	2.10 ± 1.18	5.347	518	0.000
Spatial distance	2.48 ± 1.28	1.98 ± 1.15	5.601	503	0.000
Activity frequency	2.32 ± 1.17	2.04 ± 1.10	3.274	486	0.000
Overall tolerance	2.52 ± 1.00	2.11 ± 0.89	5.871	510	0.000

**Table 6:** Age Differences in Tolerance toward Asian Elephants (Age unit: years).

Tolerance Dimension	18–30 (*N* = 63)	31–40 (*N* = 239)	41–50 (*N* = 255)	51–65 (*N* = 279)	Above 65 (*N* = 37)	f Value	df1	df2	*p* Value
Types of elephant-related damage	2.83 ± 0.96	2.68 ± 1.03	2.66 ± 1.13	2.74 ± 1.11	2.85 ± 1.01	0.632	4	868	0.639
Economic loss	2.43 ± 1.33	2.16 ± 1.35	2.31 ± 1.41	2.26 ± 1.46	2.35 ± 1.59	0.611	4	868	0.655
Population size	2.57 ± 1.29	2.39 ± 1.32	2.49 ± 1.32	2.44 ± 1.30	2.54 ± 1.50	0.360	4	868	0.837
Spatial distance	2.51 ± 1.23	2.27 ± 1.33	2.28 ± 1.18	2.39 ± 1.28	2.43 ± 1.26	0.786	4	177	0.535
Activity frequency	2.38 ± 1.18	2.25 ± 1.18	2.18 ± 1.14	2.27 ± 1.15	2.19 ± 1.27	0.454	4	868	0.769
Overall tolerance	2.54 ± 0.95	2.35 ± 0.96	2.38 ± 0.95	2.42 ± 1.02	2.47 ± 1.10	0.593	4	868	0.668

**Table 7:** Education-Level Differences in Tolerance toward Asian Elephants.

Tolerance Dimension	Primary School or Below (*N* = 460)	Junior High School (*N* = 327)	Senior High School (*N* = 57)	College or Above (*N* = 29)	f Value	df1	df2	*p* Value
Types of elephant-related damage	2.69 ± 1.07	2.71 ± 1.09	2.79 ± 1.12	3.01 ± 1.05	0.924	3	869	0.429
Economic loss	2.24 ± 1.40	2.22 ± 1.43	2.33 ± 1.31	2.97 ± 1.48	2.611	3	869	0.050
Population size	2.38 ± 1.28	2.49 ± 1.35	2.70 ± 1.36	2.83 ± 1.36	2.042	3	869	0.106
Spatial distance	2.33 ± 1.27	2.31 ± 1.27	2.40 ± 1.19	2.62 ± 1.29	0.598	3	869	0.617
Activity frequency	2.21 ± 1.13	2.28 ± 1.16	2.21 ± 1.21	2.34 ± 1.50	0.310	3	96	0.818
Overall tolerance	2.37 ± 0.96	2.40 ± 1.02	2.49 ± 0.95	2.75 ± 0.99	1.560	3	869	0.198

**Table 8:** Ethnic Differences in Tolerance toward Asian Elephants.

Tolerance Dimension	Han (*N* = 165)	Dai (*N* = 246)	Hani (*N* = 225)	Lahu (*N* = 83)	Other Ethnic Minorities (*N* = 154)	f Value	df1	df2	*p* Value
Types of elephant-related damage	2.14 ± 0.81	3.39 ± 1.05	2.57 ± 1.07	2.71 ± 1.01	2.47 ± 0.89	48.881	4	351	0.000
Economic loss	1.70 ± 1.03	3.14 ± 1.54	2.02 ± 1.26	2.01 ± 1.18	1.95 ± 1.23	34.560	4	355	0.000
Population size	2.01 ± 1.25	3.07 ± 1.37	2.30 ± 1.20	2.39 ± 1.18	2.23 ± 1.19	19.507	4	352	0.000
Spatial distance	1.62 ± 0.97	3.01 ± 1.30	2.28 ± 1.13	2.30 ± 1.18	2.13 ± 1.21	38.698	4	349	0.000
Activity frequency	1.79 ± 0.85	2.95 ± 1.30	2.00 ± 1.00	2.20 ± 1.08	1.96 ± 0.95	32.878	4	351	0.000
Overall tolerance	1.85 ± 0.60	3.11 ± 1.08	2.23 ± 0.85	2.32 ± 0.78	2.15 ± 0.78	57.187	4	356	0.000

**Table 9:** Occupational Differences in Tolerance toward Asian Elephants.

Tolerance Dimension	Wildlife Protection-Related Occupations (*N* = 111)	Other Occupations (*N* = 762)	t Value	df	*p* Value
Types of elephant-related damage	3.46 ± 1.09	2.60 ± 1.04	8.067	871	0.000
Economic loss	3.05 ± 1.61	2.15 ± 1.34	5.595	133	0.000
Population size	3.33 ± 1.35	2.33 ± 1.26	7.764	871	0.000
Spatial distance	3.18 ± 1.25	2.21 ± 1.22	7.784	871	0.000
Activity frequency	2.85 ± 1.38	2.15 ± 1.10	5.065	131	0.000
Overall tolerance	3.17 ± 1.10	2.29 ± 0.91	8.056	133	0.000

**Table 10:** Regional Differences in Tolerance toward Asian Elephants.

Tolerance Dimension	Xishuangbanna Prefecture (*N* = 446)	Pu’er City (*N* = 427)	t Value	df	*p* Value
Types of elephant-related damage	2.42 ± 0.98	3.02 ± 1.09	−8.579	871	0.000
Economic loss	1.93 ± 1.26	2.61 ± 1.48	−7.342	837	0.000
Population size	2.21 ± 1.25	2.71 ± 1.34	−5.713	860	0.000
Spatial distance	2.18 ± 1.15	2.50 ± 1.36	−3.669	835	0.000
Activity frequency	1.98 ± 0.97	2.51 ± 1.28	−6.815	792	0.000
Overall tolerance	2.14 ± 0.82	2.67 ± 1.07	−8.131	797	0.000

**Table 11:** Differences in Tolerance toward Asian Elephants by Cultivated Land Area (unit: hectare).

Tolerance Dimension	≤0.33 (*N* = 179)	0.33–0.67 (*N* = 188)	0.67–1.00 (*N* = 201)	1.00–1.33 (*N* = 164)	>1.33 (*N* = 141)	f Value	df1	df2	*p* Value
Types of elephant-related damage	2.84 ± 1.14	2.72 ± 1.11	2.70 ± 1.05	2.64 ± 1.05	2.64 ± 1.04	1.000	4	868	0.407
Economic loss	2.37 ± 1.48	2.22 ± 1.40	2.13 ± 1.33	2.33 ± 1.44	2.28 ± 1.41	0.821	4	423	0.512
Population size	2.61 ± 1.44	2.37 ± 1.30	2.35 ± 1.22	2.51 ± 1.29	2.45 ± 1.35	1.161	4	422	0.328
Spatial distance	2.56 ± 1.30	2.30 ± 1.21	2.36 ± 1.21	2.34 ± 1.33	2.06 ± 1.25	3.266	4	868	0.011
Activity frequency	2.36 ± 1.17	2.23 ± 1.18	2.15 ± 1.13	2.37 ± 1.19	2.07 ± 1.11	2.080	4	868	0.082
Overall tolerance	2.55 ± 1.05	2.37 ± 1.00	2.34 ± 0.92	2.44 ± 0.97	2.30 ± 0.95	1.723	4	868	0.143

**Table 12:** Differences in Tolerance toward Asian Elephants by Household Income (unit: thousand USD).

Tolerance Dimension	≤7.3 (*N* = 268)	7.3–14.5 (*N* = 259)	14.5–21.8 (*N* = 119)	21.8–29.1 (*N* = 135)	>29.1 (*N* = 92)	f Value	df1	df2	*p* Value
Types of elephant-related damage	2.50 ± 1.00	2.58 ± 1.08	2.58 ± 1.04	3.29 ± 1.02	3.05 ± 1.08	17.306	4	868	0.000
Economic loss	1.89 ± 1.31	2.11 ± 1.28	2.22 ± 1.28	3.11 ± 1.52	2.59 ± 1.49	17.523	4	325	0.000
Population size	2.46 ± 1.33	2.32 ± 1.29	2.24 ± 1.20	2.93 ± 1.38	2.40 ± 1.25	5.952	4	868	0.000
Spatial distance	2.08 ± 1.17	2.29 ± 1.20	2.26 ± 1.20	2.84 ± 1.40	2.57 ± 1.35	8.362	4	325	0.000
Activity frequency	2.14 ± 1.10	2.13 ± 1.07	2.06 ± 1.08	2.79 ± 1.28	2.26 ± 1.25	8.150	4	325	0.000
Overall tolerance	2.21 ± 0.88	2.29 ± 0.91	2.27 ± 0.85	2.99 ± 1.12	2.57 ± 1.04	14.176	4	325	0.000

**Table 13:** Differences in Tolerance toward Asian Elephants by Agricultural Dependence.

Tolerance Dimension	≤20% (*N* = 150)	20–40% (*N* = 159)	40–60% (*N* = 147)	60–80% (*N* = 186)	80–100% (*N* = 231)	f Value	df1	df2	*p* Value
Types of elephant-related damage	3.17 ± 1.17	2.85 ± 1.10	2.86 ± 1.00	2.56 ± 1.04	2.35 ± 0.94	16.509	4	410	0.000
Economic loss	2.81 ± 1.55	2.50 ± 1.45	2.39 ± 1.36	2.18 ± 1.39	1.73 ± 1.14	17.876	4	405	0.000
Population size	2.92 ± 1.36	2.52 ± 1.34	2.51 ± 1.23	2.33 ± 1.27	2.18 ± 1.28	8.094	4	868	0.000
Spatial distance	2.69 ± 1.32	2.43 ± 1.33	2.44 ± 1.23	2.27 ± 1.26	2.03 ± 1.13	7.168	4	410	0.000
Activity frequency	2.48 ± 1.23	2.33 ± 1.27	2.22 ± 1.10	2.18 ± 1.15	2.09 ± 1.05	2.944	4	410	0.020
Overall tolerance	2.81 ± 1.06	2.53 ± 1.05	2.48 ± 0.89	2.30 ± 0.96	2.08 ± 0.84	15.317	4	409	0.000

**Table 14:** ANCOVA of Overall Tolerance toward Asian Elephants.

Variables	f Value	df	*p* Value	Partial η^2^
Gender	21.902	1	0.000	0.025
Education Level	0.544	3	0.653	0.002
Ethnic	53.498	4	0.000	0.200
Occupation	39.062	1	0.000	0.044
Region	60.205	1	0.000	0.066
Age	0.740	1	0.390	0.001
Cultivated land area	8.696	1	0.003	0.010
Household income	4.212	1	0.040	0.005
Agricultural dependence	10.504	1	0.001	0.012
R^2^	0.374			
Adjusted R^2^	0.364			

## Data Availability

The data and materials used to support the findings of this study are available from the corresponding authors on reasonable request.

## Appendix A

**Table A1:** Parameter Estimates for the Main Significant Variables.

Variables	Reference Group	Coefficient	Standard Error	*p* Value
Han	Other ethnic minorities	−0.253	0.090	0.005
Dai		0.849	0.083	0.000
Hani		0.153	0.083	0.065
Lahu		0.297	0.109	0.007
Female	Male	−0.289	0.062	0.000
Primary school or below	College or above	−0.168	0.156	0.280
Junior high school		−0.193	0.155	0.211
Senior high school		−0.190	0.181	0.297
Other occupations	Wildlife protection-related occupations	−0.521	0.083	0.000
Pu’er City	Xishuangbanna Prefecture	0.431	0.055	0.000
Age		−0.024	0.028	0.390
Cultivated Land Area		−0.060	0.020	0.003
Agricultural Dependence		−0.066	0.020	0.001
Household Income		0.045	0.022	0.040

**Table A2:** Bonferroni Pairwise Comparisons of Overall Tolerance across Ethnic Groups.

Comparison Group	Comparison Group	Mean Difference	Standard Error	*p* Value	Sig.
Han	Dai	−1.103	0.082	0.000	Yes
	Hani	−0.407	0.082	0.000	Yes
	Lahu	−0.550	0.107	0.000	Yes
	Other ethnic minorities	−0.253	0.090	0.048	Yes
Dai	Hani	0.696	0.075	0.000	Yes
	Lahu	0.553	0.103	0.000	Yes
	Other ethnic minorities	0.849	0.083	0.000	Yes
Hani	Lahu	−0.143	0.103	1.000	No
	Other ethnic minorities	0.153	0.083	0.651	No
Lahu	Other ethnic minorities	0.297	0.109	0.068	No
